# Everyday bodily movement is associated with creativity independently from active positive affect: a Bayesian mediation analysis approach

**DOI:** 10.1038/s41598-020-68632-9

**Published:** 2020-07-20

**Authors:** Christian Rominger, Andreas Fink, Bernhard Weber, Ilona Papousek, Andreas R. Schwerdtfeger

**Affiliations:** Department of Psychology, Health Psychology Unit, University of Graz, Univ.-Platz 2, 8010 Graz, Austria

**Keywords:** Psychology, Quality of life

## Abstract

Previous (predominantly) laboratory studies reported positive relations of physical activity (or everyday bodily movement) with executive functioning, some even showed effects on creative thinking. Furthermore, positive-activated affect was found to be positively associated with everyday bodily movements and creativity. The mechanisms, however, underlying these relationships are poorly understood. The aim of this study was twofold: Firstly, we investigated whether everyday bodily movement was associated with creative performance. Secondly, we examined if positive-activated affect may mediate the association between bodily movement and creative performance. In a sample of 79 participants everyday bodily movement was recorded during five consecutive days using accelerometers. Creativity in the figural and verbal domain was assessed with performance tests, along with self-reported positive-activated affect as a trait. Findings revealed that creativity, positive-activated affect, and everyday bodily movement were associated with each other. However, positive-activated affect did not mediate the association between everyday bodily movement and creative performance. The pattern of findings argues for shared variance between bodily movement and creativity (fluency and originality) that is largely independent from variations in positive-activated affect.

## Introduction

It is well-known that physical activity has many beneficial effects on physical health. A physically active lifestyle goes along with decreases in blood pressure, protects against chronic (e.g., type 2 diabetes) and cardiovascular diseases, and decreases the risk for several types of cancer^1–3^. Beyond physical health and life expectancy^4,5^, beneficial effects of physical activity may expand to mental health (e.g., decreased depressive symptoms^6^) and psychological well-being^7–11^.

In addition, studies showed a positive association between physical activity and a broad range of cognitive skills^12–17^, however, Young and colleagues^18^ did not find an association between physical activity and cognition. Performance gains were often seen in comparatively basic types of cognitive functions, such as indicators derived from the Stroop task, or speed/accuracy measures in different attention or memory tasks^12,17^. Only a limited number of studies investigated the effects of physical activity on cognitive functions such as divergent thinking/creativity, which has indeed been found to be sensitive to physical activity interventions^19,20^.

Gondola and Tuckman^21^ were among the first who showed improved verbal creative performance (measured by means of an Alternate Uses task, AUT^22^) after participation in a running intervention^23,24^. Cavallera et al.^25^ found a positive association between the elaboration performance in a figural creative ideation task (Torrance Test of Creative Thinking, TTCT^26^) and the self-reported hours of sport activities per week (but see the work of Ramocki^27^ for a verbal creativity task). The association between self-reported physical activity and creative performance nicely corresponds to the finding that physically more fit school-aged children outperformed their less fit peers in a number of creative (and cognitive) thinking tasks^28^. Importantly, beneficial effects were not only found for chronic aspects of physical activity, such as exercise and fitness^29^, but also for more acute mechanisms accompanying (or following) bodily movement. To avoid conceptual confusion, we use the term bodily movement throughout this article when referring to daily life whole-body movements of different intensity (e.g., walking, running, but also sitting and lying). For example, Blanchette et al.^30^ found improved creative performance (in the TTCT) directly after moderate aerobic exercise^20,24,27,31,32^. Oppezzo and Schwartz^19^ found that even low-dose movement interventions (i.e., brief walking sessions) resulted in improved creative performance, however, others did not find increases after a walk or single bouts of exercise^33–36^.

While the majority of findings were based on intervention studies in controlled experimental settings (e.g., laboratory assessment, physical exercise training program^21,32^) and cross-sectional studies using a broad range of physical fitness measures^25,28^, studies using ecologically valid and objective measures of everyday physical activity are lacking. Consequently, it is still unknown if the association between physical activity and creativity generalizes to everyday life situations^37^. Therefore, the present study goes one step further and investigated this association by continuously monitoring participants’ everyday bodily movement via acceleration sensors throughout a time period of five days—including the weekend^38^. This ambulatorily assessed degree of physical activity was then correlated with the participants’ total creative performance assessed by summing up the originality of ideas of a verbal and figural creativity task (AUT, TTCT).

Although literature generally indicates a link between physical activity and creativity, only few studies investigated why physical activity is associated with creativity and addressed potential underlying mechanisms that might—at least partly—explain this relationship^20,39^. One potential factor is positive affect, which has been found to play a major role in both creativity and physical activity. The association between physical activity and positive affect is a well-established finding with small to medium effect size^7,40–42^, which was also found in ambulatory assessment studies^43,44^ (similar findings are available for arousal^32,36^). Specifically, positive-activated affect (PAA) and perceived energy seem to increase with increasing physical activity^45,46^, which was also shown in meta-analyses^40,41^.

Most important for the present investigation, Dreu and Collegues^47^ established a relationship between PAA and creative performance^48,49^. They assumed that PAA leads to higher creative fluency and originality by enhanced cognitive flexibility and reduced perseverance. Strikingly, a similar association was indicated by ecological ambulatory assessments studies reporting a simultaneous increase of positive affect and everyday creative activities^50–54^. The meta-analysis of Baas et al.^55^ estimated this correlational effect of small size (*r* = 0.08). Therefore, the second aim of the present study was to examine whether PAA may mediate the association between physical activity and creativity^17,20,47^ and therefore serve as a potential explanation of the link between physical activity and creativity.

It was firstly hypothesized, given the results of Oppezzo and Schwartz^19^ and Schwerdtfeger et al.^44^, that everyday bodily movement was associated with creativity and PAA. Secondly, given the assumption of Steinberg et al.^20^ it was hypothesized, that the association between everyday bodily movement and creativity was mediated by PAA. A significant mediation effect would indicate that PAA may at least partly explain the association between physical activity and creativity. Since specific prior evidence about the effect sizes of the correlations between physical activity, creativity, and positive affect exists, we decided for the use of Bayesian mediation analysis. This statistical approach allows incorporating prior information in the analysis and improves the efficiency of estimation and the statistical power of the analysis^56^.

## Results

### Association between everyday bodily movement and total creative performance

**Figure 1:**
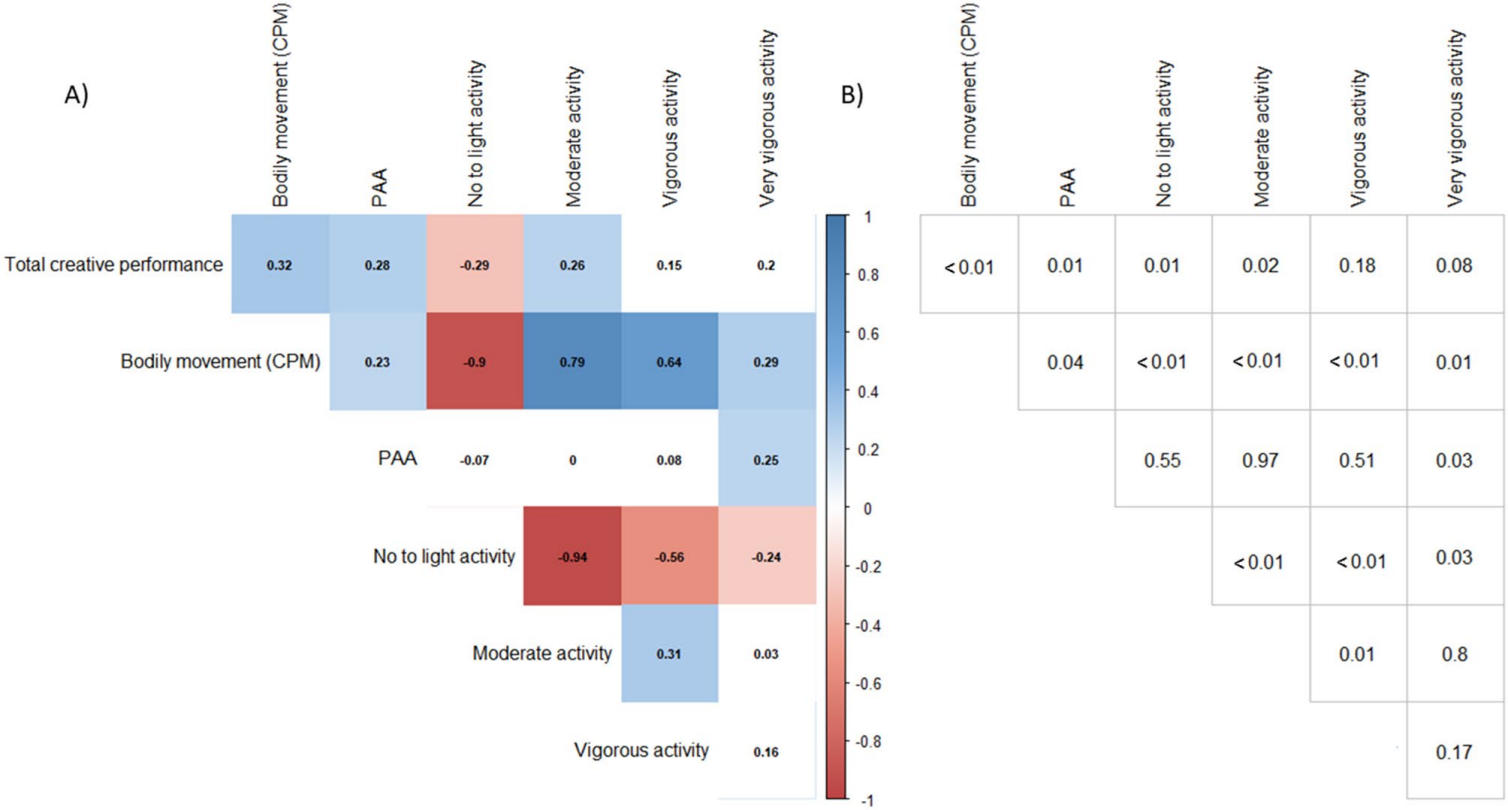
Correlation of creativity and PAA with bodily movement and the proportion of time spent with bodily movement of different intensity (i.e., no to light activity, moderate activity, vigorous activity, very vigorous activity). *PAA* positive-activated affect, *CPM* counts per minute. (**A**) illustrates the Pearson correlation coefficients and white squares are non-significant (*p* values ≥ 0.05). (**B**) shows the exact *p*-values.

In accordance with the first hypothesis, everyday bodily movement (CPM) was positively correlated with total creative performance (*r* = 0.32, *p* = 0.004). Furthermore, PAA was positively associated with both total creative performance (*r* = 0.28, *p* = 0.014) and everyday bodily movement (*r* = 0.23, *p* = 0.040; see Fig. 1).

### Mediation effect of PAA on the association between bodily movement and total creative performance

In line with the Pearson correlations, the Bayesian mediation analysis revealed that bodily movement was positively associated with both creative performance (*b* = 0.27, lower *CI* = 0.06, upper *CI* = 0.49) and PAA (*b* = 0.23, lower *CI* = 0.01, upper *CI* = 0.46). These findings are depicted in Table 1.

**Table 1:** Bayesian mediation analysis.

	Estimate (*SE*)	l-95% CI	u-95% CI	Effective sample	Rath
Total creative performance (intercept)	0.00 (0.11)	− 0.21	0.21	24,908	1.00
PAA (intercept)	0.00 (0.11)	− 0.22	0.22	23,482	1.00
Bodily movement (CPM)—total creative performance	0.27 (0.11)	0.06	0.49	21,405	1.00
Bodily movement (CPM)—PAA	0.23 (0.11)	0.01	0.46	26,751	1.00
Total creative performance—PAA	0.21 (0.11)	− 0.01	0.44	24,868	1.00

*PAA* positive-activated affect, *CPM* counts per minute, *CI* credible intervals.

*SE* in parenthesis.

The total effect of the mediation model was *b* = 0.32 (lower *CI* = 0.10, upper *CI* = 0.54) and the indirect effect was *b* = 0.04 (lower *CI* = -0.02, upper *CI* = 0.13). The indirect effect added only 13.51% (lower *CI* = -12.85%, upper *CI* = 39.87%) to the direct effect of bodily movement on creative performance (*b* = 0.27, lower *CI* = 0.06, upper *CI* = 0.49). This argues against a strong mediating effect of PAA on the relationship between bodily movement and creativity, since the *CIs* include a negative percentage. Due to the absence of a clear mediation, the pattern of findings indicates that activity and PAA are independently linked with creative performance (see Table 1). These results remained virtually unchanged when including age, gender, and BMI as covariates.

### Additional analyses

#### Proportion of time spent with different types of bodily movement

In order to evaluate the association of physical activity with creative performance and PAA in more detail, exploratory correlation analyses were calculated with the proportion of time spent with bodily movements of four different intensity levels. As illustrated in Fig. 1, total creative performance showed significant positive associations with time spent in moderate (e.g., walking) and a negative association with movements of no to light intensity (e.g., sitting and lying). However, PAA was associated with time spent with very vigorous physical activity, but not with the proportion of time spent with sedentary behaviour or movement at low and moderate intensities. This pattern of findings indicate that the variance of CPM associated with PAA might be qualified by very vigorous activity.

#### Originality and fluency of creative performance

Since the measure of total creative performance has been criticized due to its high correlation with the fluency of ideas^57–59^, we also investigated, which aspects of creativity (originality, fluency) were linked with bodily movement. The regression analysis showed that both originality (*sr* = 0.23, *p* = 0.038) and fluency (*sr* = 0.25, *p* = 0.024) were positively and independently from each other associated with physical activity. The significant regression analysis indicated that the quantity and the quality of ideas together shared 9.6% of variance with physical activity (*F*(2,76) = 4.06, *p* = 0.021). A similar regression analysis investigating the association of fluency and originality with PAA slightly failed to show significance (*F*(2,76) = 3.00, *p* = 0.056). Only fluency (*sr* = 0.27, *p* = 0.018), but not originality (*sr* = 0.10, *p* = 0.354) was associated with the PAA. Originality (*r* = 0.52, *p* < 0.001) and fluency (*r* = 0.70, *p* < 0.001) were correlated with total creative performance.

## Discussion

This study examined firstly, whether everyday bodily movement is associated with creativity and secondly, if this association is mediated by participants’ PAA. Importantly, this study replicated the reported positive relationship between bodily movement and creativity, which to date was predominantly found in laboratory studies^21,25^. We assessed bodily movement in everyday life by means of acceleration sensors. In contrast to former methods, the applied ecological assessment methodology objectively measures the level of spontaneous bodily movement in daily life—free from systematic errors of subjective self-reports^38^.

A more detailed examination of bodily movement (i.e., categorization into different intensity levels) indicated that not only time spent with activities of high intensity, like sport and bouts of exercise, go along with increased creative performance^21,28,30^, but also everyday physical activities of a moderate intensity level^19^. The negative relationship between the proportion of time spent with light intensities—including sedentary behavior—and creativity further underlines the a priori assumption that physical activity and bodily movement have beneficial effects on creative cognition^19^. This is also nicely in accordance with the anecdotal view that creative people use bodily movement to overcome mental blocks and lacks of inspiration^20,32,60,61^ (but see^62^).

Bodily movement was also linked to PAA^40,41^, which in turn was associated with creativity^35,47,55^. Importantly and critically, while all three variables of interest were interrelated, the Bayesian mediation analysis indicated that PAA did not explain the relationship between bodily movement and creativity. This finding corresponds to a former study. Steinberg et al.^20^ reported an impact of physical activity on creative performance and positive affect. However, they did not find evidence for a mediation. Importantly, together with Steinberg et al.^20^, the present finding suggests an additive effect of PAA and physical activity on creativity. This conclusion may also have important consequences: (1) Other psychological mechanisms have to be considered responsible for the observed relationship between physical activity and creativity. It might be hypothesized that the personality trait openness to experiences may serve as a potentially mediating variable, because it goes along with creativity and physical activity^63,64^. (2) To promote creativity, the induction of PAA plus the engagement in physical activity separately might be more fruitful in contrast to one strategy alone. This is in line with the observation that PAA was mainly associated with the fluency of ideas, but bodily movement seemed to be linked with the quantity and quality of ideas.

In accordance with this, Curnow and Turner^39^ reported an additional effect in a music (i.e., arousal) and exercise combined group compared to exercise and music alone groups in TTCT fluency, but they did not find differences in originality. However, this study investigated acute effects of exercise and emotional arousal on creativity, while the present study focused on the chronic aspect of this relationship, thus hampering a direct comparison of results. To overcome this limitation of solely focusing on chronic or acute mechanisms in future studies, experience-sampling methods (i.e., ecological momentary assessment of everyday life behavior), which enable the measurement of creativity, affective states, and bodily movement in the very same moment of time, should be applied^44,65^. This innovative approach may allow a more detailed insight into the mechanisms responsible for the relationship between physical activity and creativity. In conjointly using ecological momentary assessment and multi-level analyses, the chronic as well as acute effects of physical activity (and positive affect) on creative performance may be distinguished.

While in accordance with previous research higher PAA was associated with more bodily movement^41,44–46^ the more detailed analyses—using the proportion of time spent with bodily movement at different intensity levels—showed a divergent pattern of correlations. PAA was linked with very vigorous physical activity, but not with moderate and low intensities^40,42^. These additional analyses indicate one further explanation for the absence of a mediation effect. While a sedentary lifestyle seems to be associated with lower creativity scores, it shows no association with the affect. With other words: Because affect and creativity are associated with different intensity levels of everyday bodily movement, movement associated changes in affect might therefore be independent from movement associated changes in creativity. This conclusion is well in line with an additive effect of affect and physical activity on creativity. Despite the promises of ecological momentary assessment studies as outlined above, the present study was interested to assess the trait component of all variables and tested whether variance of the proposed mediator (i.e., PAA) overlaps with the shared variance of the predictor (i.e., bodily movement) and the criterion (i.e., creative performance).

This study is not without limitations. It is important to note that—due to the cross-sectional study design—the reported correlations are not causal in nature. Hence, it would also be possible, for instance, that creativity leads to more positive affect^65^ and higher levels of positive affect result in more everyday bodily movement^44^. Due to the moderate (and even low) reliability of the measures of bodily movement, PAA, and creativity, results should be treated with caution. However, physical activity was assessed on five different days including the weekend. Thus, a moderate internal consistency is well in line with the assumed variations of bodily movement throughout a week^66^. Similarly, total creative performance was assessed by two measures in two domains (i.e., figural and verbal), further decreasing internal consistency, which was in the reported range of various scoring methods in this field^59^. Although, the total creative performance score showed the expected association with bodily movements, it has also been criticized^57–59,67^, and a differentiation between quality (i.e., originality) and quantity (i.e., fluency) of ideas has been suggested. In accordance with the total creative performance score, originality and fluency were positively associated with bodily movement, however, only fluency was associated with PAA. This is in line with many laboratory studies investigating the association with physical activity by means of creativity measures combining fluency and originality of ideas^19,28,30^. Finally, the sample size of the present study might be too small to detect a small effect of PAA on the association between creativity and physical activity by means of a frequentist approach. However, the use of informative priors in a Bayesian mediation analysis increases the power of analyses and the precision of estimates^56^. Nevertheless, and not at least due to the high number of correlations in this study, a replication of the findings, also by means of a momentary ecological assessment approach is recommended before far-reaching conclusions are warrantable.

To sum up, the present findings are in accordance with the common notion that physical activity has many beneficial effects. Besides the well-known effects on physical health, a greater degree of everyday bodily movement is associated with higher levels of PAA (and subjective well-being^40^) and better creative cognition^12,19^. This study represents a first step to investigate this complex relationship in everyday life. The findings argue for an association between everyday bodily movement and creativity that is not merely attributed to the PAA. Further studies are certainly warranted to elucidate other pathways.

## Methods

### Participants

Seventy-nine participants (42 women) with an age range from 18 to 33 years (*M* = 22.95; *SD* = 3.34) took part in the study. Their Body Mass Index (BMI) varied between 17.29 kg/m^2^ and 39.18 kg/m^2^ (*M* = 22.81 kg/m^2^, *SD* = 3.43 kg/m^2^). All participants were university students. The highest degree of completed education ranged from job that requires training (1.3%) to academic degree (26.6%; grammar school, 56.9%; vocational school, 15.2%). Participants with chronic or acute musculoskeletal impairment were not included in the sample. The study was approved by the ethics committee of the University of Graz (approval number GZ. 39/21/63 ex 2015/16), methods were carried out in accordance with this, and written informed consent was obtained.

### Creativity tasks

#### Verbal creativity

In the Alternate Uses (AU) task^22^ participants had to produce as many original and creative uses of conventional everyday objects (e.g., umbrella, car tire) as possible within 1 min each. In total participants worked on five items. This procedure resulted in 1,164 separable ideas in total. The originality of each idea was rated by three instructed and independent raters on a four-point rating scale ranging from 1 (not original at all) to 4 (highly original; cf. Consensual Assessment Technique^68^; see, e.g., Rominger et al.^69^). The inter-rater agreement was ICC (2, k) = 0.75. Participants’ verbal creative performance was indexed by the sum of all originality scores of the produced ideas (*M* = 44.36, *SD* = 12.05; similar procedure see^20,26,70^). Since studies reported a high correlation between this additive scoring procedure of creative performance and the fluency of ideas^57,58,71^, the quantity (i.e., fluency, mean number of answers per item constituted the individual fluency score, *M* = 4.91, *SD* = 1.27) and quality (i.e., originality, average of all originality scores of a participant; *M* = 1.82, *SD* = 0.26) of ideas were calculated for additional analyses.

#### Figural creativity

In the Picture Completion Task of the Torrance Test of Creative Thinking (TTCT^26^) participants have to extend abstract lines and figures in an original and creative way. Similar to the procedure of the AU task, the originality of each picture was evaluated by three independent raters on a four-point scale ranging from 1 to 4 (ICC (2, k) = 0.79)^72^. The raters were instructed to rate the originality of the idea only, and not the drawing skills or the title of the drawing. Participant’s figural creative performance score was calculated as the sum of all produced originality scores, that is a weighted sum (*M* = 18.03, *SD* = 3.79; similar procedure see ^20,26,70^). Fluency was determined as the number of completed pictures within the given time-limit of 10 min (734 pictures in total; *M* = 9.29, *SD* = 1.21). Originality was indicated as the average of all originality scores of a participant (*M* = 1.95, *SD* = 0.35).

#### Total creative performance

In order to indicate participant’s total creative performance, the z-transformed verbal and figural creative performance scores were aggregated (α = 0.45). For additional analyses the fluency and originality scores were calculated by the sum of the z-transformed scores. Cronbach’s alpha for fluency was α = 0.63 and α = 0.59 for originality.

#### Objective measurement of everyday bodily movement

Bodily movement was recorded by means of tri-axial acceleration sensors (ActiGraph®, Model GT3X + , weight of 19 g). All participants carried the sensor on the right side of their hip, attached with an elastic belt^66,73,74^. This approach provides a measure of metabolically relevant whole-body movements and their intensity levels^66^. The acceleration data were sampled with 30 Hz and activity counts were analyzed using 1 min epochs (i.e., counts/min, CPM; see Schwerdtfeger et al.^44^ for a similar approach). The activity counts were the composite vector magnitude of the three axes^75,76^. The CPM as the main output of the ActiGraph^77^ was validated in a high number of studies (e.g.,^78^; for details how ActiGraph counts are generated, see Brønd, Andersen, & Arvidsson^79^). All data were analyzed with the software ActiLife (Version 6.13.2).

The acceleration data were recorded on five consecutive days during waking hours, in order to reliably and representatively measure the degree of participants’ everyday bodily movement^66,80^. The recordings started on Wednesday 00:00 and ended on Sunday at 23:59. Since all participants were instructed to note the sensors’ non-wearing times in a calendar template (e.g., during bathing, or sleeping), we were able to exclude invalid periods of sensor data (i.e., counts of zero) from CPM estimation. The minimum of wearing time per day was set to 600 min (*M* = 839.89 min, *SD* = 79.69 min)^38^. Non-activities longer than 90 min during the self-rated wearing time were treated as invalid non-activities and all counts of zero during this time were excluded from the CPM estimation^81^. The resulting data were finally quality checked for spurious counts (i.e., CPMs above 20,000 were inspected in more detail)^80,82^. This procedure resulted in *M* = 4.99 (*SD* = 0.01) valid days per participant (max = 5, min = 4) showing a mean CPM of 601.38 (*SD* = 168.54). There was no significant difference of CPM between the five days of measurement (*F*(4,74) = 1.367; *p* = 0.245). The reliability and the validity of the applied method are acceptable^66,83^ with a Cronbach’s α of 0.61 in the present study (for all five days of recording).

In addition to CPM as an index of general everyday bodily movement, the proportion of minutes (relative to the total wearing time) spent in no to light (< 2,690 CPMs; *M* = 93.60%, *SD* = 2.84%), moderate (2,690–6,166 CPMs; *M* = 5.40%, *SD* = 2.43%), vigorous (6,167–9,642 CPMs; *M* = 0.76%, *SD* = 0.77%), and very vigorous activity (> 9,642 CPMs; *M* = 0.26%, *SD* = 0.50%) was analyzed. These measures are estimates of the probability with which participants show movement in a specified intensity level. The classification cut-points were in accordance with former studies^76^.

#### Positive-activated affect (PAA)

Positive-activated affect as a trait was assessed by four items of the German version of the Positive and Negative Affect Schedule (PANAS)^84,85^. Participants were requested to indicate how they feel in general on all 20 adjectives of the PANAS on a five-point Likert scale (from 1 = not at all to 5 = very much so). Similar to Schwerdtfeger and Gerteis^86^, PAA was indexed by the mean of the four items^87^ (“active”, “alert”, “attentive”, “inspired”; *M* = 3.47, *SD* = 0.55, Cronbach’s α = 0.61).

### Procedure

All participants were tested separately. Firstly, they signed the informed consent, followed by the PANAS and the divergent thinking tests (AUT, TTCT). Participants were familiarized with the study protocol and the technical equipment (acceleration sensor, belt). Following the instruction, the accelerometer was initialized and attached. Finally, participants were instructed to report the sensor wearing times in a template. After the objective measurement of habitual bodily movement, participants returned the sensors and finally rated the typicality of the recording days on a four-point rating scale (1 = absolutely typical, 2 = rather typical, 3 = rather untypical, 4 = totally untypical). No participant rated these days as totally untypical (17.72% absolutely typical, 69.62% rather typical, 12.66% rather untypical).

### Statistical analysis

In order to answer the first study question, Pearson correlation was used to test the relationship between everyday bodily movement and total creative performance. Furthermore, as an important prerequisite for a mediation, the inter-correlations of all three variables of interest (i.e., CPM, PAA, and total creative performance) were calculated^88^.

Bayesian mediation analysis was applied to evaluate if the association between everyday bodily movement and creativity was due to the influence of PAA. The statistical program R (version 3.4.3)^89^ with the package brms (version 2.7.0)^90^ was used. A Bayesian approach instead of a common frequentist approach was applied, because of (1) the moderate sample size of 79 participants^56,91^ and (2) the a priori knowledge about the intercorrelations. The use of prior information in Bayesian statistics increases the power of the applied mediation analysis^56^. Firstly, physical activity is associated with increased creativity^19,21^. Secondly, physical activity is positively associated with PAA^44^, and, thirdly, PAA is linked with creativity^47,55^. Based on relevant literature, normally distributed informative priors for the regression coefficients of bodily movement and creativity (*µ* = 0.12, σ^2^ = 0.10; Etnier et al.^12^ reporting a *d* of 0.25), bodily movement and PAA (*µ* = 0.27, σ^2^ = 0.10; Reed & Buck^40^ reporting a *d* of 0.57), and creativity and PAA (*µ* = 0.08, σ^2^ = 0.10)^55^ were used^56^.

Samples were derived by Markov Chain Monte Carlo (MCMC) algorithm, 4 chains with 5,000 iterations (1,000 warm-up samples for each chain) and 16,000 post-warmup samples were used. Unstandardized effect estimates (b), 95% credible intervals (CI), and the potential scale reduction factor on split chains (Rhat) are reported. For Rhat, values close to 1 indicate that the chains converged (i.e., the number of iterations was sufficient). To evaluate the mediating role of PAA on the association between bodily movement and creativity the direct, indirect, and total effect, and the effect proportion of the mediation are reported. This was done with the R package mediation (version 4.4.6)^92^. The mediating effect of PAA is considered as present, when the credible intervals (*CIs*) of the indirect effect are positive and do not include zero.

In additional analyses the association between the intensity levels of physical activity and total creative performance with the proportion of time spent with physical activities in different intensity levels were calculated. Equivalent correlations were calculated for PAA. Since the total creative performance score has been criticized to be mainly driven by the fluency of ideas^58,93^, additional analyses were calculated to investigate the association of originality and fluency with bodily movements, by means of a regression analysis. This approach allows to investigate the unique variance quality and quantity of ideas share with physical activity. A similar regression analysis was calculated for PAA.

## Data Availability

The data will be available from the first author.
